# Effects of Adding MedX Lumbar Extension or Deadlift Exercises to a Core Stabilization Program on Extensor Strength, Disability, and Pain in Men with Chronic Low Back Pain: A Randomized Pilot Trial

**DOI:** 10.3390/healthcare14162550

**Published:** 2026-08-14

**Authors:** Jin Woong Park, Wi-Young So, Jae Keun Oh, Dong Chun Lim

**Affiliations:** 1 Sports Medicine Laboratory, Graduate School, Korea National Sport University, Seoul 05541, Republic of Korea; qkrwlsdnd325@gmail.com; 2 Department of Sports Medicine, College of Humanities, Korea National University of Transportation, Chungju 27469, Republic of Korea; wowso@ut.ac.kr

**Keywords:** activities of daily living, chronic low back pain, extensor exercise, MedX, pain index

## Abstract

**Background and Objectives:** Despite extensive research on the management of chronic low back pain, evidence regarding the additional benefits of incorporating lumbar extension-specific exercises into conventional core stabilization programs remains limited. Therefore, the primary objective of this pilot study was to evaluate the feasibility of three 8-week exercise interventions in men in their 30s with chronic low back pain, including recruitment feasibility, retention rate, adherence to exercise sessions, intervention acceptability, and safety. The secondary and exploratory objective was to determine preliminary intervention effects and variability parameters for lumbar extensor strength, activities of daily living, and pain intensity. These findings may help in designing, selecting the outcome, and calculating sample size of a future definitive randomized controlled trial with adequate statistical power. **Methods:** The study included 30 male participants aged 30–39 years with low back pain persisting for more than 12 weeks. They were randomly divided into a MedX Exercise Program Group, a Deadlift Exercise Program Group, and a Core Exercise Program Group. The intervention consisted of exercise sessions conducted twice weekly for eight weeks. The intervention was evaluated by measuring lumbar extensor strength, the ability to perform activities of daily living, and pain intensity before and after the intervention. The collected data were tested for normality using the Shapiro–Wilk test, and homogeneity was tested using one-way analysis of variance. Group differences between time periods were tested using two-way analysis of variance with repeated measures. For outcome variables showing significant between-group differences at baseline, additional analyses of covariance were conducted with the corresponding baseline values included as covariates. Effect sizes and 95% confidence intervals were calculated and reported for all outcome variables. As this pilot study was not designed to provide confirmatory efficacy testing, analyses of secondary outcomes were considered exploratory in nature, and no adjustments for multiple comparisons were applied. **Results:** All 30 participants who underwent eligibility screening were randomized, and all participants completed the 8-week intervention and follow-up assessments, resulting in a 100% retention rate. Intervention adherence was also 100%, with all 480 scheduled sessions completed (480/480). No intervention-related adverse events were reported, and complete data were available for all outcome variables. For lumbar extensor strength, a significant main effect of time was observed across all seven lumbar extension angles (all *p* < 0.001), indicating significant improvements within all three groups. A significant time × group interaction was observed only at 0° (*p* = 0.048); however, subsequent pairwise comparisons revealed no significant differences between any groups at follow-up. Activities of daily living significantly improved in all three groups, and no significant between-group differences were observed in the baseline-adjusted analysis of variance (*p* = 0.226). Pain intensity significantly decreased in all groups, with the largest reduction observed in the core stabilization exercise group (−78.05%). Post-hoc analyses demonstrated that the reduction in pain intensity was significantly greater in the core stabilization exercise group than in both the MedX lumbar extension exercise group (*p* = 0.022) and the deadlift exercise group (*p* = 0.042). **Conclusions:** All three 8-week exercise interventions were feasible, achieving complete participant retention and adherence, with no reported adverse events. Although lumbar extensor strength, activities of daily living, and pain intensity significantly improved across all groups, no additional benefits were noted from incorporating MedX lumbar extension exercises or deadlift exercises into a common core stabilization exercise program. Notably, the greatest reduction in pain intensity was observed in the core stabilization exercise-only group. Given the exploratory nature and pilot design of this study, these findings should be interpreted cautiously and require confirmation in future adequately powered randomized controlled trials.

## 1. Introduction

In contemporary society, rapid industrialization has intensified workloads, and inadequate physical activity and poor nutritional habits have contributed to increasing obesity rates [1]. Additionally, the growing prevalence of sedentary lifestyles has caused weakening of the lumbar vertebral musculature, thereby increasing the incidence of low back pain [2,3]; consequently, it is classified as a major disease alongside joint and heart diseases [4]. Furthermore, it is one of the most frequently reported conditions in orthopedic practice worldwide [2,3,4,5].

Low back pain induces functional impairment in the musculature and surrounding soft tissues of the lumbar region, thereby reducing muscular strength, muscular endurance, and flexibility [6]. The incidence of low back pain increases markedly from the age of 30 years [7], and it frequently occurs in the absence of identifiable causes. Clinically, it is classified according to symptom duration: pain persisting for less than 6 weeks is defined as acute pain, pain lasting 6–12 weeks is considered subacute pain, and pain continuing for more than 12 weeks is regarded as chronic pain. Notably, chronic low back pain is closely associated with reduced quality of life [6].

Chronic low back pain may be categorized as pain attributable to specific spinal pathologies—such as a herniated intervertebral disc—or pain without an identifiable cause. The participants in this study were adult men who used community exercise facilities and had experienced low back pain for more than 12 weeks but had not been tested or diagnosed to exclude specific spinal pathologies. Therefore, the study population should not be interpreted as being limited to individuals with non-specific low back pain. Rather, it should be regarded as a cohort of individuals with chronic low back pain of undetermined etiology. Individuals with chronic low back pain often exhibit reduced engagement in activities of daily living (ADLs), and such functional limitations may further exacerbate pain [8]. Although symptoms of low back pain are observed across a wide age spectrum, from childhood to individuals in their 60s and beyond [2,4], insufficient education regarding appropriate spinal care and obesity-related factors may increase the chances of progression from acute to chronic low back pain [4,5,6,9].

The risk of low back pain increases with advancing age. Psychosocial factors—such as anxiety and depression associated with psychological stress—also contribute to an elevated risk [9,10]. Findings on spinal extensor strength and chronic low back pain indicate that weakness in lumbar extensor muscles is a critical risk factor [11,12]. Specifically, reduced spinal extensor strength exacerbates pain and serves as a primary contributor to the persistence of pain among individuals with chronic low back pain. For individuals with such symptoms, rehabilitation exercise programs are broadly categorized into two types: lumbar extensor strengthening exercises and core stabilization exercises [13].

The MedX lumbar extension exercise helps alleviate back pain by stabilizing the pelvis and lower extremities, thereby limiting compensatory movements from other muscle groups and allowing for the isolated activation of the lumbar extensor muscles [14]. Similarly, the deadlift, a well-known weight-training exercise for developing the posterior chain, is associated with reduced low back pain and improved spinal extensor strength when appropriately prescribed [15]. Notably, recent studies have demonstrated that core stabilization exercise programs that incorporate flexibility training are effective in reducing pain among individuals with chronic low back pain [16]. Participants with this condition typically exhibit reduced spinal extensor strength; therefore, spinal extension exercises and core stabilization exercises are essential components of their rehabilitation [16].

Recently published systematic reviews and meta-analyses have synthesized the evidence regarding the effectiveness of these exercise interventions for chronic low back pain. A 2025 meta-analysis of isolated lumbar extension exercises included eight randomized controlled trials involving 381 participants and demonstrated a significant reduction in pain intensity compared with untreated controls (effect size [ES] = −0.633, *p* = 0.004). However, no significant improvements were observed in functional disability (ES = −0.292, *p* = 0.190) or isometric lumbar extensor strength (ES = 0.967, *p* = 0.150), and the overall certainty of the evidence was rated as very low [17]. Importantly, because of the limited number of studies employing active comparator groups, a separate pooled analysis of active-controlled trials could not be conducted. Consequently, the available evidence does not support the conclusion that isolated lumbar extension exercises provide superior clinical benefits compared with other exercise interventions.

A separate meta-analysis of posterior chain resistance exercises, including deadlift-based training, analyzed eight studies involving 408 participants and found that both posterior chain resistance exercises and general exercise programs improved outcomes related to chronic low back pain. However, the between-group differences became more evident following longer intervention periods (12–16 weeks) than after shorter ones (6–8 weeks) [18]. Similarly, a 2025 meta-analysis comparing Pilates, core stabilization training, and core resistance training reported that all three interventions significantly improved pain and physical function, with no significant differences between exercise modalities (*p* = 0.24) [19]. Furthermore, a network meta-analysis comparing multiple exercise interventions concluded that core stabilization and stabilization-based exercise programs significantly improved pain and physical function in individuals with chronic low back pain. Nevertheless, the authors noted that the current evidence is insufficient to recommend one exercise modality over another because of the limited quality and quantity of the available studies [20].

Collectively, these findings indicate that lumbar extension exercises, core stabilization exercises, and deadlift-based resistance training are all effective interventions for chronic low back pain. However, no study has established the superiority of any single exercise modality, and direct comparisons using active control groups remain scarce. Moreover, because most previous studies have evaluated each exercise modality as a standalone intervention, evidence regarding the incremental benefits of incorporating lumbar extension-specific exercises or deadlift training into a standardized core stabilization program remains limited.

More recently, several studies have begun to investigate the additive effects of combining other exercise modalities with core stabilization programs. Kuzu et al. [21] randomly assigned 60 older adults with chronic non-specific low back pain to either a core stabilization exercise group or a combined intervention group that performed aerobic exercise in addition to core stabilization exercises. Following an 8-week intervention, both groups demonstrated significant improvements; however, the combined intervention group achieved greater improvements in functional capacity, physical performance, fall risk, and pain intensity. These findings suggest that, although core stabilization exercise is effective as a standalone intervention, combining it with complementary exercise modalities may provide additional clinical benefits. Nevertheless, evidence from such combination-based intervention studies remains limited. In particular, the potential additive effects of incorporating resistance-based exercises, such as lumbar extension-specific exercises or deadlift training, into a core stabilization program have not yet been adequately investigated.

However, limited research exists comparing the effectiveness of core stabilization programs that utilize MedX lumbar extension exercises with those that incorporate the deadlift. Accordingly, it becomes imperative to determine which type of extension-based exercise is more effective in improving spinal extensor strength and pain outcomes in participants with chronic low back pain. However, studies directly comparing the relative effects of adding MedX lumbar extension or deadlift exercises to a standardized core stabilization program remain scarce. Moreover, before conducting a fully powered randomized controlled trial, it is necessary to establish the feasibility of these interventions and generate preliminary estimates of their potential effects. Accordingly, this pilot study had two primary objectives. Objective 1 was to evaluate the feasibility of an 8-week intervention incorporating either MedX lumbar extension exercises or deadlift exercises into a common core stabilization program by assessing recruitment, retention, attendance compliance, intervention acceptability, data completeness, and safety. Objective 2 was to characterize changes in lumbar extensor strength, activities of daily living, and pain intensity across the three intervention programs, and to generate preliminary estimates of effect sizes, confidence intervals, and outcome variability to inform the design and sample size calculation of a future definitive trial. Because this pilot study was not designed to establish treatment efficacy or determine the superiority of one exercise modality over another, the analyses were conducted from an exploratory perspective rather than through formal hypothesis testing.

## 2. Materials and Methods

### 2.1. Design and Participants

This pilot study was designed as a prospective, randomized, single-blind controlled trial. The study followed a two-time-point design (pre- and post-intervention). Baseline assessments were conducted three days before the intervention, and post-intervention assessments were performed, on average, three days after its completion. The study participants were recruited from individuals who visited a Medi&Fit fitness center in Seoul, Republic of Korea. Participants included only men aged 30–39 years with chronic low back pain who had experienced symptoms for at least 12 weeks. The age range was limited to individuals in their 30s based on epidemiological evidence indicating that the prevalence of low back pain increases substantially from the age of 30 onward. This age group was selected to evaluate the effects of exercise intervention in individuals at a stage when chronic low back pain begins to become clinically established. The study was further restricted to male participants to minimize biological heterogeneity. Sex-related differences exist in both the absolute magnitude of lumbar extensor muscle strength and the patterns of adaptation to resistance exercise. Therefore, in a pilot study with only 10 participants per group, inclusion of both sexes was considered likely to increase outcome variability and reduce the precision of the preliminary estimates. Furthermore, national physical activity surveys have reported that participation in moderate- to vigorous-intensity physical activity is approximately 25% among men and 15% among women [22], suggesting that the impact of insufficient physical activity on chronic low back pain may differ by sex. These eligibility restrictions were implemented as a deliberate design strategy to maximize sample homogeneity and internal validity in this small-scale pilot study. However, this may limit the external validity and generalizability of the findings. This issue is addressed in the Section 4.2.

Eligibility was limited to those who, after receiving a comprehensive explanation of the study’s purpose and procedures, voluntarily expressed their willingness to participate. Participants were men aged 30–39 years who self-reported low back pain persisting for at least 12 weeks. Participants were recruited from community exercise facilities, and specific spinal pathologies, such as intervertebral disc herniation or spinal stenosis, were neither screened for nor excluded through imaging examinations or clinical diagnoses. Therefore, the study participants should not be considered a sample of individuals with non-specific chronic low back pain, but rather a population with chronic low back pain of undetermined etiology. Participants were excluded if they had any limitation that prevented accurate assessment of lumbar extensor muscle strength, had undergone surgical or non-surgical treatment for low back pain within the previous 3 months, or reported a pain intensity score of ≥5 points.

Recruitment was conducted through posted notices on the center’s bulletin boards as well as verbal announcements. Exclusion criteria included limitations in assessing lumbar extensor muscle strength, having undergone either non-surgical or surgical treatment within the preceding three months, and a pain intensity score of five or higher on a numeric rating scale. Because this study was designed as a pilot and feasibility trial, a formal power analysis for confirming intervention efficacy was not considered an appropriate primary basis for sample size determination. Instead, the sample size was determined according to methodological recommendations for pilot studies [23,24,25], with 10 participants per group and a total sample size of 30 participants. Nevertheless, to ensure transparency and reproducibility, all parameters of the preliminary power analysis conducted during the initial study design phase are reported. The analysis was performed using G*Power version 3.1.9.7 (Heinrich-Heine-University, Düsseldorf, Germany) [26]. The selected test was “ANOVA: Repeated measures, within–between interaction” under the F-test family, with the following assumptions: effect size f = 0.50, α = 0.05, statistical power = 0.80, three groups, two measurement time points, a correlation of 0 between repeated measurements, and a nonsphericity correction ε = 1. Under these assumptions, the estimated minimum sample size was 24 participants. Therefore, 30 participants were recruited to account for an anticipated 20% dropout rate. Three aspects of this calculation warrant clarification. First, the calculation was not based on a specific clinical outcome variable; rather, it represented a general estimation for detecting a group × time interaction effect in a three-group, two-time-point design. Furthermore, no primary efficacy outcome was prespecified because the present study was not designed to test treatment efficacy. In the current manuscript, the primary outcome is the feasibility assessment, whereas lumbar extensor strength, activities of daily living, and pain intensity are considered secondary exploratory outcomes. Second, the assumed effect size (f = 0.50) was not derived from previous studies or preliminary data but was selected as a conventional value representing a large effect according to Cohen’s criteria. Third, the assumption of zero correlation between repeated measurements represents a conservative approach compared with the correlations typically observed in pre–post intervention studies, thereby yielding a larger estimated sample size. However, the use of an assumed large effect size without empirical justification may have resulted in an underestimation of the sample size required for a definitive efficacy trial. This issue is acknowledged as a limitation of the present pilot study.

**Figure 1:**
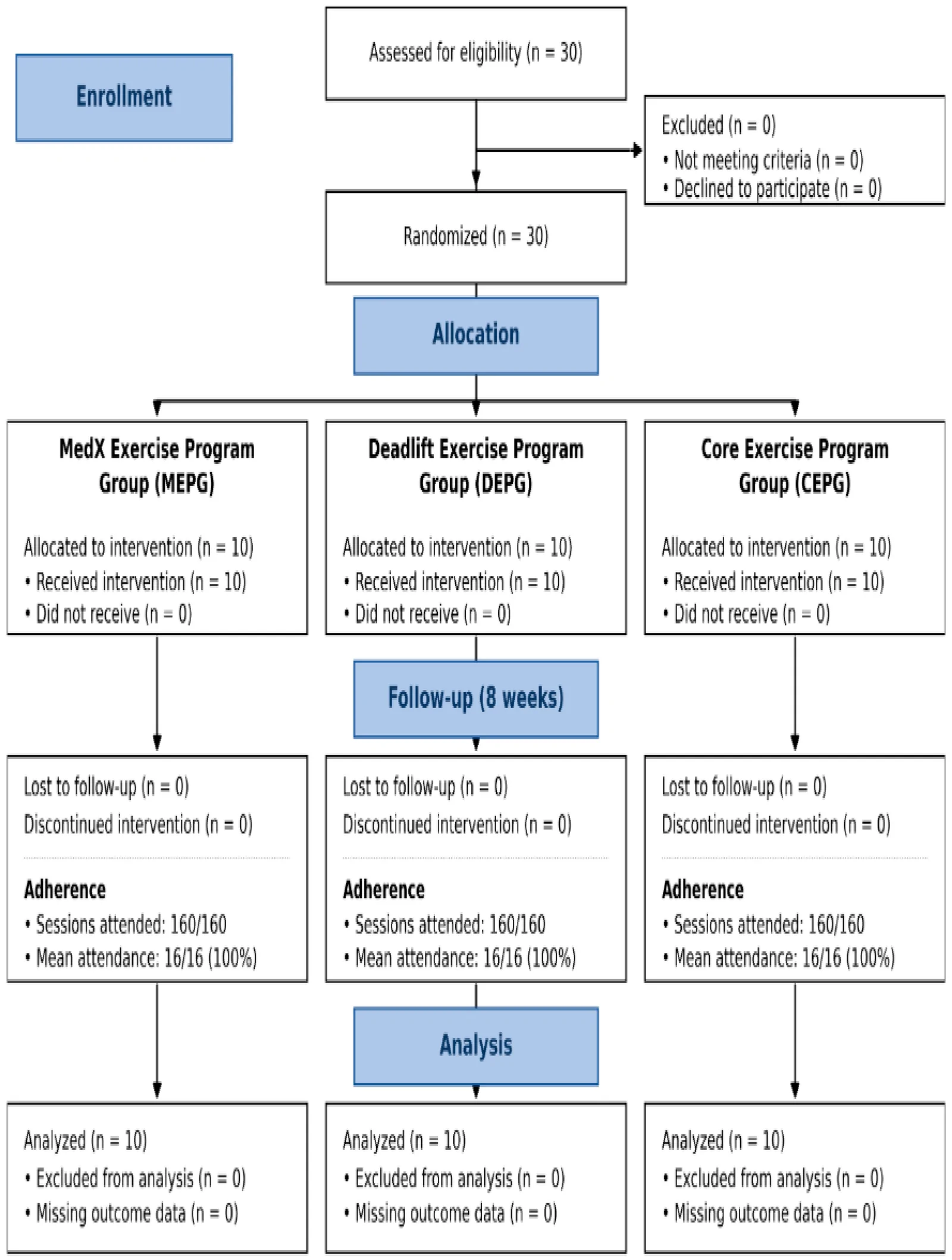
CONSORT flow diagram. Adherence was defined as the number of sessions attended out of the 16 scheduled sessions (twice weekly for 8 weeks). All 30 participants attended all 16 sessions (480/480 sessions, 100%).

These 30 participants were randomly distributed into three groups: the MedX Exercise Program Group (MEPG; *n* = 10), the Deadlift Exercise Program Group (DEPG; *n* = 10), and the Core Exercise Program Group (CEPG; *n* = 10). The CONSORT flow diagram applied in this study is presented in Figure 1. The baseline characteristics of the participants and the baseline values of the outcome variable are presented in Table 1. The results of the between-group tests for baseline characteristics are intended to describe the participants’ starting points and are not presented as evidence to prove the validity of randomization. This study was designed as a prospective experimental study conducted in a non-clinical setting. All procedures in this study were approved by the Institutional Review Board of Korea National Sport University (IRB approval number: 1263-202406-HR-084-02; approval date: 19 July 2024) and were conducted in accordance with the principles of the Declaration of Helsinki. Prior to IRB approval, only the identification of potential participants who expressed an interest in participating in the study was performed. During this period, no written informed consent was obtained, no eligibility assessments were conducted, and no research-related data were collected. Following IRB approval, potential participants were contacted, provided with detailed information regarding the study objectives and procedures, and asked to provide written informed consent. Eligibility screening was conducted only after informed consent had been obtained. The first participant was enrolled on 22 July 2024. Data collection, including the 8-week intervention and pre- and post-intervention assessments, was completed on 20 September 2024, and the study was concluded on 27 September 2024. This study was retrospectively registered with the Clinical Research Information Service (CRiS) of the Korea Disease Control and Prevention Agency on 7 June 2026, after completion of participant enrollment (registration number: KCT0012086). The potential methodological implications of retrospective registration, including concerns regarding selective outcome reporting and reduced transparency compared with prospective registration, are addressed in Section 4.2. Additionally, the participants were informed that they could withdraw from the study at any time during the study period.

#### 2.1.1. Randomization and Blinding

The 30 participants who met the eligibility criteria were randomly allocated to three groups (10 participants per group): the MedX lumbar extension exercise group (MEPG), deadlift exercise group (DEPG), and core stabilization exercise group (CEPG). However, because the study protocol was not prospectively registered prior to participant recruitment, the specific method used to generate the randomization sequence, the personnel responsible for the randomization process, and the implementation of allocation concealment procedures were not documented at the time of the study and could not be verified retrospectively. Therefore, the adequacy of allocation concealment cannot be confirmed. Blinding of participants and intervention providers was not feasible because of the nature of the exercise interventions. However, pre- and post-intervention outcome assessments were conducted by an independent researcher who was not involved in group allocation. Furthermore, the researcher responsible for intervention delivery and outcome assessment was not involved in statistical analysis. Therefore, the study incorporated assessor blinding, although participant and intervention-provider blinding were not possible. The absence of documented randomization procedures and the inability to verify allocation concealment introduce the potential for selection bias. This limitation should be considered when interpreting baseline differences between groups, including the observed imbalance in ADLs scores. This issue is addressed in the Section 4.2.

#### 2.1.2. Feasibility Outcome Variables and Progression Criteria

The primary outcomes of this study consisted of five feasibility indicators: (i) recruitment: the enrollment ratio relative to the number of eligible individuals and the time required to complete recruitment; (ii) retention rate: the proportion of participants who completed the post-intervention assessment; (iii) attendance compliance: the number of attended sessions out of the 16 scheduled sessions and the proportion of participants who attended ≥75% of the sessions; (iv) intervention acceptability: the number of participants who requested withdrawal or discontinued participation due to intervention-related reasons; and (v) safety: the number and severity of intervention-related adverse events. The secondary outcomes, including lumbar extensor strength, activities of daily living, and pain intensity, were considered exploratory outcomes intended to generate preliminary estimates of effect sizes, confidence intervals, and outcome variability for the planning of a future definitive trial, rather than to provide confirmatory evidence of intervention efficacy. The progression criteria were defined as follows: Go was defined as a retention rate of ≥80%, Amend as a retention rate of 60–79%, and Stop as a retention rate of <60%. Attendance compliance was considered acceptable when participants attended at least 75% of the 16 scheduled sessions, and progression required the absence of serious intervention-related adverse events. However, because the study protocol and statistical analysis plan were not prospectively registered prior to participant recruitment, these progression criteria were established retrospectively and should not be interpreted as prespecified decision rules. This limitation is acknowledged in Section 4.2. The reporting of this study followed the CONSORT 2010 extension for randomized pilot and feasibility trials [27].

### 2.2. Exercise Intervention

Each group underwent an eight-week exercise program as the intervention. All three groups in this study received the same core stabilization exercise program. In addition, the MEPG performed MedX lumbar extension exercises, whereas the DEPG performed deadlift exercises. Therefore, this study employed an add-on design to examine the incremental effects of incorporating different primary exercise modalities into a standardized core stabilization program, rather than comparing exercise interventions with a non-exercise control condition. All groups participated in supervised exercise sessions twice weekly for 8 weeks. The duration of each session differed according to the intervention group: sessions for the MEPG and DEPG lasted approximately 45 min because they included the additional main exercise components, whereas sessions for the CEPG lasted approximately 35 min because no additional main exercise was performed. The structure and duration of the core stabilization program were identical across all groups, consisting of a 15-min warm-up, 15 min of bracing and core stabilization exercises, and a 5-min cool-down. All exercise interventions were performed at a Medi&Fit fitness center in Seoul, Republic of Korea, under standardized equipment and supervision conditions, and were conducted in a non-clinical setting rather than in a hospital or clinical institution.

#### 2.2.1. MedX Exercise Program

The MedX exercise program was conducted using MedX 96 (Ocala, FL, USA). We assessed the maximal lumbar extensor strength and set the training intensity at 40–50% of this maximum. The procedure for adjusting exercise intensity throughout the intervention period is described in Section 2.2.4. For the warm-up, participants performed stretching exercises targeting the hip region for 10 s per muscle group across two sets, for a total duration of 15 min. Regarding strengthening exercises, participants performed lumbar extension exercises in three sets of 15 repetitions, totaling approximately 10 min. As additional exercises, participants performed bracing maneuvers in supine and standing positions, holding each for 10 s across 20 repetitions. Core exercises were performed in two sets of 15 repetitions, for a total duration of 15 min. Finally, a 5-min cycling session was included as the cool-down (Table 2).

#### 2.2.2. Deadlift Exercise Program

The deadlift exercise program was adapted and refined based on previously established protocols [28]. After conducting an indirect estimation of the one-repetition maximum, we set the training intensity at 40–50% of this maximum [28]. The procedure for adjusting exercise intensity throughout the intervention period is described in Section 2.2.4. For the warm-up, participants performed stretching exercises targeting the hip region for 10 s per muscle group across two sets, for a total duration of 15 min. Regarding strengthening exercises, participants performed Romanian deadlifts in three sets of 15 repetitions, totaling 10 min. As additional exercises, bracing was performed in supine and standing positions, maintaining each for 10 s and repeating 20 times. Core exercises were performed in two sets of 15 repetitions, for a total duration of 15 min. Finally, the cool-down phase consisted of 5 min of cycling (Table 3).

#### 2.2.3. Core Exercise Program

The core exercise program consisted solely of warm-up and additional exercises. The core exercise program group performed only the core stabilization exercises that were common to all three intervention groups and did not receive any additional primary exercise modality, such as MedX lumbar extension or deadlift exercises. Therefore, this group should be considered an active comparison group receiving the standardized core stabilization program alone, rather than a non-exercise control group with no exercise intervention. The warm-up comprised stretching exercises targeting the hip region, performed for 10 s per muscle group across two sets, for a total duration of 15 min. The additional exercises included bracing maneuvers executed in supine and standing positions, holding each for 10 s and repeating 20 times in each position. Core exercises were also performed in two sets of 15 repetitions, totaling 15 min. Finally, a 5-min cycling session was performed as a cool-down exercise [16] (Table 4).

#### 2.2.4. Supervision, Intensity Control, and Compliance with Intervention

Equivalence of Exercise Volume: All three groups performed the same warm-up (15 min), core stabilization exercises (two sets of 15 repetitions; approximately 15 min), and cool-down (5 min of cycling). The only difference between groups was the inclusion and type of the additional primary exercise modality. Specifically, the MEPG performed MedX lumbar extension exercises, and the DEPG performed Romanian deadlift exercises (three sets of 15 repetitions; approximately 10 min), whereas the CEPG did not perform an additional primary exercise. Consequently, the total session duration was approximately 45 min for the MEPG and DEPG and approximately 35 min for the CEPG. Although the common exercise components and their volume were identical across groups, the total exercise volume differed because of the presence or absence of the additional exercise component. This difference was an inherent feature of the add-on design, which was intended to evaluate the incremental effects of incorporating specific additional exercises into a standardized core stabilization program. This issue is addressed in the Section 4.2.

Supervision: All exercise sessions were conducted under the supervision of exercise instructors affiliated with the facility where the study was conducted. Instructors were present throughout each session to monitor exercise performance, provide corrective feedback regarding movement technique, and instruct participants to discontinue the relevant exercise if pain or discomfort occurred.

Intensity Adjustment: All groups performed the intervention for 8 weeks using the initial exercise intensity established at the beginning of the program. The initial intensity was set at 40–50% of maximal lumbar extension strength for the MEPG and at 40–50% of the estimated one-repetition maximum based on an indirect maximal repetition assessment for the DEPG. During the intervention period, exercise intensity was individually adjusted by supervisors based on the perceived exertion and exercise performance of the participants. However, predefined criteria for progression, including the timing and magnitude of intensity increases, were not established, and systematic reassessments of maximal strength or maximal repetition capacity were not performed during the 8-week intervention. This approach was adopted to prioritize safety within a low-intensity exercise protocol for individuals with chronic low back pain. Nevertheless, the absence of standardized progression criteria is acknowledged as a limitation.

Compliance: The intervention consisted of 16 supervised sessions conducted twice weekly over 8 weeks. All 30 participants completed all 16 sessions, resulting in an attendance compliance rate of 100% (480 attended sessions out of 480 scheduled sessions). No make-up sessions were required, and no participants requested intervention discontinuation or withdrew from the study. Rest intervals between sets and movement velocity were not prespecified in the intervention protocol and were not recorded during the intervention period. Therefore, these parameters could not be reported in the present manuscript. The lack of documentation regarding these variables is acknowledged as a limitation affecting intervention reproducibility.

### 2.3. Outcome Measures

#### 2.3.1. Lumbar Extensor Strength

Lumbar extensor strength was assessed using the MedX Lumbar Extension Machine (MedX 96, Ocala, FL, USA) in accordance with the MedX testing protocol. Before testing, participants performed approximately 10 min of preparatory warm-up stretching and received sufficient instruction regarding the equipment and testing procedure. However, a separate session for familiarizing them with the measurement equipment was not conducted in this study. Instead, participants received detailed instructions and performed warm-up trials on the day of the pre-intervention assessment. During the assessment, the pelvis and thigh were fully stabilized using pelvic and thigh restraint systems, while a posterior head pad was applied to ensure proper alignment. Participants grasped the handles with both hands throughout the procedure. Testing was conducted at lumbar flexion angles of 72°, 60°, 48°, 36°, 24°, 12°, and 0°, with maximal isometric lumbar extension strength measured once at each angle in a sequential manner. Participants maintained forward gaze during testing and received visual feedback, while maximal effort was exerted against the backrest for 1–3 s at each interval to achieve peak force output at each angular position. Between testing intervals, participants performed lumbar extensor stretching and rested for 20–30 s. A single maximal isometric contraction was performed at each lumbar flexion angle in accordance with the standardized MedX testing protocol. This approach was adopted because repeated maximal contractions across seven different angles could introduce cumulative fatigue, potentially leading to a systematic underestimation of strength measurements at subsequent angles. The measurement sequence was standardized for all participants and conducted in a descending order of flexion angle (72° to 0°). To minimize the influence of fatigue, lumbar extensor stretching and a 20–30 s rest period were provided between measurements at each angle. However, because repeated measurements were not obtained and mean values from multiple trials were not calculated, the reliability and within-participant variability of the measurements could not be estimated. This issue is acknowledged as a limitation of the present study. Lumbar extensor strength was recorded in foot-pounds (ft-lb). For statistical analyses, maximal isometric extensor strength values obtained at each flexion angle were analyzed separately. The summed strength value across all measured angles was not calculated or included as an outcome measure. The reliability of the MedX Lumbar Extension Machine has been reported as 0.93 [29].

#### 2.3.2. Ability to Perform ADLs

ADLs were assessed using the ODI (Oswestry Disability Index), a widely used instrument for evaluating functional disability associated with low back pain during daily activities. The validated Korean version of the ODI was used in this study [30]. The questionnaire consists of 10 items assessing pain intensity, personal care, lifting, walking, sitting, standing, sleeping, sexual activity, social life, and traveling. Each item is scored from 0 to 5 points, resulting in a total raw score ranging from 0 to 50 points, with higher scores indicating greater functional disability. In the present study, raw ODI scores (0–50 points) were used for statistical analyses. However, because ODI scores are commonly reported internationally as percentages (0–100%), the corresponding percentage scores were also presented in the results table for comparability with previous studies. Percentage scores were calculated as follows: (raw ODI score ÷ 50) × 100.

#### 2.3.3. Pain Intensity

Low back pain intensity was assessed using an 11-point numeric rating scale (NRS) ranging from 0 to 10, where 0 indicates no pain and 10 represents the worst pain imaginable. Participants were asked to select and report a single integer that best represented their current pain intensity at the time of assessment [31]. Unlike the visual analog scale (VAS), which requires participants to indicate their pain intensity by marking a point along a continuous line, the NRS used in this study requires the selection of a discrete numerical value from 0 to 10. Therefore, this measure was appropriately classified as a numeric rating scale rather than a visual analog scale.

### 2.4. Data Analysis

Descriptive statistics, such as means and standard deviations, were used to present the data. Normality was assessed using the Shapiro–Wilk test, and the sample was found to satisfy the assumption of normality (*p* > 0.05). Homogeneity among the groups was examined using one-way analysis of variance. Baseline comparability between groups was examined using one-way analysis of variance (ANOVA) for both demographic characteristics and baseline values of all outcome variables, with the results presented in Table 1. However, these baseline comparisons were not conducted to determine whether randomization was successful. When randomization is properly implemented, baseline differences are expected to occur by chance, and statistical significance testing of baseline characteristics does not provide evidence regarding the validity of group allocation. Therefore, baseline comparisons in this study were performed solely to describe participant characteristics at study entry and to identify outcome variables with baseline imbalances that may require covariate adjustment.

Two-way repeated-measures ANOVA was performed to examine the main effects of time, group, and the time × group interaction. For outcome variables showing significant baseline differences between groups, repeated-measures ANOVA alone was considered insufficient to adequately account for baseline imbalance. Therefore, additional analyses using analysis of covariance (ANCOVA), with baseline values entered as covariates, were conducted. Adjusted means and Bonferroni-corrected post hoc pairwise comparisons were reported. Prior to ANCOVA, the assumption of homogeneity of regression slopes was evaluated by testing the group × covariate interaction term. All statistical results were reported with F-values, degrees of freedom, and exact *p*-values. *p*-values were presented to three decimal places, with values < 0.001 reported as *p* < 0.001. Effect sizes were expressed as partial eta squared (ηp^2^) with corresponding 95% confidence intervals, which were calculated using the noncentral F distribution. For within-group pre–post changes, mean differences, 95% confidence intervals, and Cohen’s dz values were reported.

Regarding statistical assumptions, the within-subject factor in the repeated-measures analyses consisted of only two time points (pre-intervention and post-intervention). Therefore, the assumption of sphericity was not applicable. When a repeated-measures factor contains only two levels, the variance of the difference score has only one estimate, and no comparison among variances is possible; consequently, Mauchly’s test of sphericity cannot be performed and corrections such as Greenhouse–Geisser adjustment are unnecessary. The assumption of homogeneity of variances between groups was assessed using Levene’s test. Although this assumption was satisfied for most outcome variables, unequal variances were observed for changes in lumbar extensor strength at 0° and 12° flexion angles and for post-intervention activities of daily living scores. However, because all groups had equal sample sizes (*n* = 10 per group), the F-test is considered relatively robust to moderate violations of variance homogeneity. This issue is acknowledged in the Section 4.2. Observed power was not calculated in this study. Because observed power is derived by treating the observed effect size as the true population parameter, it is mathematically dependent on the *p*-value and does not provide additional information beyond the hypothesis test itself [32,33,34]. Therefore, confidence intervals were reported to provide information regarding the precision and uncertainty of the effect estimates.

Because this study was designed as an exploratory pilot study, statistical analyses of secondary outcomes were conducted primarily for hypothesis generation rather than confirmatory inference. Repeated-measures ANOVA was performed for nine exploratory outcomes, including lumbar extensor strength measured at seven angles, activities of daily living, and pain intensity. Therefore, the potential inflation of Type I error due to multiple testing should be considered. Two approaches were adopted to address this issue. First, adjustment for multiple testing across different outcome domains was not applied in the primary analyses. This decision was based on the exploratory nature of the pilot study, in which the primary objective was to generate preliminary effect estimates rather than confirm specific hypotheses. Applying overly conservative corrections at this stage may increase the risk of excluding potentially meaningful signals for future definitive trials. Accordingly, *p*-values were interpreted as descriptive rather than confirmatory, and greater emphasis was placed on effect sizes and 95% confidence intervals. Second, to evaluate the potential influence of multiple testing, a sensitivity analysis was conducted by treating the nine outcome variables as a single family of tests. The results of Bonferroni, Holm, and Benjamini–Hochberg adjustments are presented in Appendix A (Table A1). In addition, Bonferroni correction was applied to post hoc pairwise comparisons following significant time × group interactions (Table A2). The standard deviations of changes in each outcome variable were also reported to provide preliminary estimates for sample size calculation in future definitive trials. All analyses were performed using SPSS Statistics 25.0 (IBM Corp., Armonk, NY, USA), with statistical significance set at *p* < 0.05.

## 3. Results

### 3.1. Feasibility Outcomes

Recruitment began immediately after Institutional Review Board (IRB) approval (19 July 2024), using a previously established list of individuals who had expressed interest in participating in the study. The first participant was enrolled on 22 July 2024. All 30 participants who underwent eligibility assessment met the inclusion criteria and were randomly allocated to one of the three groups (10 participants per group), with no exclusions after screening. No participants failed to receive their allocated intervention, were lost to follow-up, or discontinued the intervention. Therefore, the retention rate was 100% (30/30). Attendance compliance was also 100%, with all participants completing all 16 scheduled sessions (480 attended sessions out of 480 scheduled sessions), and all 30 participants (100%) meeting the predefined attendance criterion of attending at least 75% of the sessions. Data completeness was 100%, as all participants were included in the final analysis and no missing data were observed for any outcome variables. No participants requested withdrawal due to intervention-related reasons. However, a formal system for prospectively collecting and documenting adverse events was not established in this study. During the intervention period, no adverse events were reported by participants or observed by the supervising instructors. According to the retrospectively established progression criteria, the retention rate, attendance compliance, and data completeness outcomes met the predefined thresholds for progression.

### 3.2. Changes in Lumbar Extensor Strength at 0°

Table 5 presents the results concerning lumbar extensor strength at a lumbar flexion angle of 0°. The main effect of time was significant (F(1, 27) = 68.894, *p* < 0.001, ηp^2^ = 0.718, 95% CI [0.494, 0.813]), indicating significant changes over time across all groups. The time × group interaction was also significant (F(2, 27) = 3.397, *p* = 0.048, ηp^2^ = 0.201, 95% CI [0.000, 0.405]). However, the main effect of group was not significant (F(2, 27) = 1.821, *p* = 0.181, ηp^2^ = 0.119). Bonferroni-adjusted post hoc pairwise comparisons following the significant time × group interaction revealed no statistically significant differences between any group pairs (MEPG vs. DEPG: mean difference = 45.30, *p* = 0.073; MEPG vs. CEPG: mean difference = 29.30, *p* = 0.455; DEPG vs. CEPG: mean difference = −16.00, *p* = 0.852).

### 3.3. Changes in Lumbar Extensor Strength at 12°

Table 6 presents the results concerning lumbar extensor strength at a lumbar flexion angle of 12°. The main effect of time was significant (F(1, 27) = 75.297, *p* < 0.001, ηp^2^ = 0.736, 95% CI [0.521, 0.824]), indicating significant changes over time across all groups. However, neither the main effect of group (F(2, 27) = 1.952, *p* = 0.162, ηp^2^ = 0.126) nor the time × group interaction (F(2, 27) = 1.278, *p* = 0.295, ηp^2^ = 0.086) was statistically significant.

### 3.4. Changes in Lumbar Extensor Strength at 24°

Table 7 presents the results concerning lumbar extensor strength at a lumbar flexion angle of 24°. The main effect of time was significant (F(1, 27) = 85.599, *p* < 0.001, ηp^2^ = 0.760, 95% CI [0.560, 0.840]), indicating significant changes over time across all groups. However, neither the main effect of group (F(2, 27) = 1.868, *p* = 0.174, ηp^2^ = 0.122) nor the time × group interaction (F(2, 27) = 0.524, *p* = 0.598, ηp^2^ = 0.037) was statistically significant.

### 3.5. Changes in Lumbar Extensor Strength at 36°

Table 8 presents the results for lumbar extensor strength at a lumbar flexion angle of 36°. The main effect of time was significant (F(1, 27) = 94.418, *p* < 0.001, ηp^2^ = 0.778, 95% CI [0.589, 0.852]), indicating significant changes over time across all groups. However, neither the main effect of group (F(2, 27) = 1.945, *p* = 0.163, ηp^2^ = 0.126) nor the time × group interaction (F(2, 27) = 0.862, *p* = 0.434, ηp^2^ = 0.060) was statistically significant.

### 3.6. Changes in Lumbar Extensor Strength at 48°

Table 9 presents the results for lumbar extensor strength at a lumbar flexion angle of 48°. The main effect of time was significant (F(1, 27) = 82.125, *p* < 0.001, ηp^2^ = 0.753, 95% CI [0.548, 0.835]), indicating significant changes over time across all groups. However, neither the main effect of group (F(2, 27) = 1.677, *p* = 0.206, ηp^2^ = 0.110) nor the time × group interaction (F(2, 27) = 1.503, *p* = 0.240, ηp^2^ = 0.100) was statistically significant.

### 3.7. Changes in Lumbar Extensor Strength at 60°

Table 10 presents the results concerning lumbar extensor strength at a lumbar flexion angle of 60°. The main effect of time was significant (F(1, 27) = 118.201, *p* < 0.001, ηp^2^ = 0.814, 95% CI [0.651, 0.876]), indicating significant changes over time across all groups. However, neither the main effect of group (F(2, 27) = 1.855, *p* = 0.176, ηp^2^ = 0.121) nor the time × group interaction (F(2, 27) = 1.904, *p* = 0.168, ηp^2^ = 0.124) was statistically significant.

### 3.8. Changes in Lumbar Extensor Strength at 72°

Table 11 presents the results for lumbar extensor strength at a lumbar flexion angle of 72°. The main effect of time was significant (F(1, 27) = 93.257, *p* < 0.001, ηp^2^ = 0.775, 95% CI [0.585, 0.851]), indicating significant changes over time across all groups. The main effect of group was not significant (F(2, 27) = 1.488, *p* = 0.244, ηp^2^ = 0.099). The time × group interaction did not reach statistical significance (F(2, 27) = 3.058, *p* = 0.064, ηp^2^ = 0.185).

### 3.9. Changes in the Ability to Perform ADLs

Changes in the ability to perform ADLs are presented in Table 12. The main effects of time (F(1, 27) = 261.816, *p* < 0.001, ηp^2^ = 0.907, 95% CI [0.818, 0.938]), group (F(2, 27) = 3.782, *p* = 0.036, ηp^2^ = 0.219, 95% CI [0.000, 0.422]), and the time × group interaction (F(2, 27) = 7.754, *p* = 0.002, ηp^2^ = 0.365, 95% CI [0.067, 0.548]) were statistically significant. However, because a significant baseline difference in ADLs scores was observed among groups (F(2, 27) = 6.079, *p* = 0.007, ηp^2^ = 0.310), an additional analysis of covariance (ANCOVA) was performed with baseline ADLs scores entered as covariates. Following adjustment for baseline values, the main effect of group was no longer significant (F(2, 26) = 1.574, *p* = 0.226, ηp^2^ = 0.108). The adjusted mean ADLs scores were 8.98 for the MEPG, 10.33 for the DEPG, and 8.09 for the CEPG. Bonferroni-adjusted post hoc pairwise comparisons revealed no significant differences among any of the group comparisons (MEPG vs. DEPG, *p* = 0.276; MEPG vs. CEPG, *p* = 0.575; DEPG vs. CEPG, *p* = 0.414). Therefore, although significant between-group differences were observed in the initial repeated-measures analysis, these differences were not maintained after adjustment for baseline imbalance.

### 3.10. Changes in Pain Intensity

Table 13 presents the results for pain intensity. The main effect of time (F(1, 27) = 152.488, *p* < 0.001, ηp^2^ = 0.850, 95% CI [0.714, 0.900]), the main effect of group (F(2, 27) = 7.971, *p* = 0.002, ηp^2^ = 0.371), and the time × group interaction (F(2, 27) = 5.861, *p* = 0.008, ηp^2^ = 0.303, 95% CI [0.028, 0.497]) were all statistically significant. Because no significant baseline differences in pain intensity were observed among the groups (F(2, 27) = 0.079, *p* = 0.924), an additional analysis of covariance (ANCOVA) was not performed. Bonferroni-adjusted post hoc pairwise comparisons following the significant time × group interaction revealed that the reduction in pain intensity was significantly greater in the CEPG than in both the MEPG (mean difference = −1.40, *p* = 0.022, Hedges’ *g* = −1.30) and the DEPG (mean difference = −1.30, *p* = 0.042, Hedges’ *g* = −1.17). No significant difference was observed between the MEPG and DEPG (mean difference = −0.10, *p* = 1.000, Hedges’ *g* = −0.10).

## 4. Discussion

This pilot study aimed to evaluate the feasibility of an 8-week intervention incorporating either MedX lumbar extension or deadlift exercises into a common core stabilization program and to generate preliminary estimates for a future definitive trial involving men in their 30s with chronic low back pain. Regarding feasibility outcomes, all three programs demonstrated excellent retention and attendance compliance, with no reported intervention-related adverse events. These findings support the feasibility and acceptability of implementing a fully powered randomized controlled trial. However, findings related to the secondary outcome variables should be interpreted as exploratory. Given the limited sample size and the primary purpose of this pilot study, the present results were not designed to establish the efficacy or superiority of any specific intervention. Therefore, the observed changes should be considered preliminary estimates to inform the design, sample size calculation, and outcome selection for a future definitive trial.

Lumbar extensor strength constitutes a critical determinant of spinal health [35]. Prior research has demonstrated that strengthening both the lumbar extensors and the abdominal musculature is essential for preventing low back pain [35]. Comparative analyses between participants with low back pain and asymptomatic healthy individuals have revealed that participants experiencing low back pain exhibit reduced activation of the spinal extensor muscle groups [35,36]. Additionally, individuals experiencing chronic low back pain tend to report substantially lower lumbar muscle strength compared with healthy counterparts. Intervention studies have reported that lumbar extension strength training induces marked improvements in extensor strength and significantly reduces pain among individuals with chronic low back pain [37,38]. These findings suggest that enhancing lumbar stability is essential for preventing low back pain and helps improve overall quality of daily life [35,36,37,38].

In this study, lumbar extensor strength was measured using the MedX Lumbar Extension Machine, which enables isolation of the lumbar musculature by stabilizing the pelvis and lower extremities, while minimizing the involvement of other muscle groups. Furthermore, the machine enables the assessment of lumbar extensor strength across seven distinct angular positions, facilitating precise measurement at each angle. The findings indicate that MedX-based lumbar extension training permits accurate, angle-specific quantification of extensor strength and enables the prescription of individualized exercise intensity based on these measurements. This capacity for tailored intervention is considered to have contributed to the substantial improvements observed, consistent with the findings of previous studies [39,40]. Park and Lee [39] reported that continuous lumbar extension training enhances spinal stability, thereby improving extension capacity. Another study found that MedX training is effective because it isolates lumbar extensor function without interference from other muscle groups, provides a fixed and stable body position without lateral deviation, and restricts movement to a precisely controlled range of motion [40]. In summary, these findings suggest that lumbar extension-specific exercises may contribute to improvements in lumbar extensor muscle strength among men in their 30s with chronic low back pain. However, although improvements in extension capacity were observed in this study, the effects on flexion capacity were not assessed. Therefore, further research is warranted to investigate the impact of MedX lumbar extension training on spinal flexor strength. However, this study did not provide statistical evidence supporting the superiority of MedX lumbar extension exercises over the other exercise programs. A significant time × group interaction was observed only at the 0° angle (*p* = 0.048); however, none of the post hoc pairwise comparisons between groups reached statistical significance (minimum *p* = 0.073), and the finding did not remain significant after adjustment for multiple comparisons (Bonferroni-adjusted *p* = 0.435). At the remaining six angles, only significant main effects of time were observed. These findings indicate that all three groups demonstrated significant improvements in lumbar extensor muscle strength over time; however, there was insufficient evidence to conclude that the magnitude of improvement differed among the exercise programs. This pattern is consistent with recent meta-analytic evidence. A meta-analysis of lumbar extension-specific resistance exercise reported significant reductions in pain intensity compared with untreated control conditions but found no significant improvements in isometric muscle strength. Furthermore, the effects of lumbar extension-specific exercise may not differ substantially from those of other exercise modalities when evaluated against active comparison groups [17]. The present study employed an active comparison design in which all groups performed the same core stabilization program, with MedX lumbar extension exercises or deadlift exercises added as supplementary interventions. Therefore, the absence of significant between-group differences may reflect the comparable effects of different exercise approaches when a common active exercise component is provided. Additionally, a meta-analysis of posterior chain resistance exercises reported that differences between exercise approaches were more apparent in longer intervention periods (12–16 weeks) than in shorter ones (6–8 weeks) [18]. Accordingly, the 8-week intervention period used in the present study may have been insufficient to detect potential additional effects of MedX lumbar extension exercises beyond the common core stabilization program.

In this study, the ODI was employed to assess functional disability associated with chronic low back pain. The results revealed a statistically significant reduction in the level of functional disability across all groups following the intervention. Two plausible explanations may account for these findings. First, MedX lumbar extension training stabilizes the pelvis and lower extremities while restricting the involvement of non-target musculature, thereby enabling isolated activation of the lumbar extensors in a controlled and safe manner. Additionally, exercise intensity can be individualized based on prior assessment of one’s extension capacity. As reported in previous studies, lumbar extension training might have lowered disability scores related to daily activities [41]. Second, core stabilization programs yield significant improvements in functional disability among affected individuals [42]. Collectively, these findings suggest that MedX-based lumbar extension training and core stabilization exercises are effective for improving the ability to perform ADLs among participants with low back pain. However, baseline ADLs scores differed significantly among the groups (F(2, 27) = 6.079, *p* = 0.007). The DEPG had a lower baseline ADLs score than the other two groups, indicating a potentially limited capacity for further improvement due to lower initial impairment. Therefore, the significant group effect observed in the repeated-measures ANOVA may have reflected baseline differences rather than true intervention effects. Supporting this interpretation, when baseline ADLs values were included as covariates in ANCOVA, the between-group difference was no longer statistically significant (F(2, 26) = 1.574, *p* = 0.226).

The potential influence of regression to the mean should also be considered in relation to this baseline imbalance. For ADLs, the groups with higher baseline impairment (MEPG and CEPG) demonstrated larger reductions, whereas the group with lower baseline impairment (DEPG) showed smaller changes. Therefore, regression to the mean may have contributed partially to the observed pattern of ADLs improvement. In contrast, baseline pain intensity did not differ among groups (F(2, 27) = 0.079, *p* = 0.924), suggesting that regression to the mean alone cannot explain the greater pain reduction observed in the CEPG. Thus, regression to the mean should be considered when interpreting ADLs outcomes but does not sufficiently explain the pain-related findings.

The absence of between-group differences in ADLs despite significant improvements in lumbar extensor strength across all groups highlights the multidimensional nature of functional disability in chronic low back pain. Canli and Özüdoğru [43] reported that disability in patients with chronic nonspecific low back pain is influenced by multiple independent factors, including activity-related pain, fear of movement, lower-extremity muscle strength, balance ability, lumbar mobility, functional status, spinal posture, and spinal control, which collectively explained a substantial proportion of the variance in ODI scores. Therefore, functional disability in chronic low back pain is unlikely to be determined solely by lumbar extensor muscle strength but rather reflects interactions among physical, psychological, and functional domains.

From this perspective, the comparable improvements in ADLs among the three groups may be explained by the fact that all participants performed core stabilization exercises and engaged in regular supervised exercise participation. These factors may have improved functional disability through mechanisms beyond muscle strength enhancement, including improved motor control, reduced fear of movement, and increased confidence in physical activity. Future studies should consider incorporating biopsychosocial measures, such as fear of movement, psychological distress, and balance ability, to better elucidate mechanisms underlying functional improvement. An unexpected finding of this study was that the CEPG, which performed only core stabilization exercises, demonstrated the greatest reduction in pain intensity. Although all three groups showed significant reductions in pain (MEPG: −42.86%; DEPG: −46.34%; CEPG: −78.05%), the reduction in the CEPG was significantly greater than that in the MEPG (mean difference = 1.40, *p* = 0.022, Hedges’ *g* = 1.30) and DEPG (mean difference = 1.30, *p* = 0.042, Hedges’ *g* = 1.17). Because this finding was contrary to the original hypothesis, several possible explanations should be considered.

First, the analgesic effects of core stabilization exercises themselves may have contributed to this finding. Core stabilization exercise is an established intervention for chronic low back pain and has been reported to improve neuromuscular control of deep trunk musculature, enhance spinal stability, and reduce pain [16,44]. In this study, the CEPG performed the same core stabilization program as the other groups without additional lumbar loading exercises. Therefore, it is possible that these participants experienced sufficient therapeutic effects while experiencing less exercise-induced fatigue or delayed muscle soreness. Conversely, MedX lumbar extension exercises and deadlift exercises impose greater mechanical demands on the lumbar extensor musculature, which may enhance strength adaptation but potentially influence short-term pain perception through fatigue or muscle soreness.

Second, the effects of general exercise participation should be considered. Previous research has suggested that regular physical activity itself, rather than a specific exercise modality alone, substantially alleviates chronic low back pain. The significant pain reductions observed in all three groups suggest that consistent participation in supervised exercise over eight weeks may have contributed to symptom improvement.

Third, contextual and psychological factors may have influenced pain outcomes. Because participant and intervention-provider blinding was not possible, expectancy effects, attention from supervisors, and other contextual influences associated with exercise participation cannot be excluded. However, there is no evidence that these factors selectively favored the CEPG. Indeed, the opposite pattern would have been expected if novelty or equipment-based expectations had played a dominant role, as the MedX group received a more specialized intervention.

Additionally, this study did not monitor analgesic use or concomitant treatments during the intervention period. Therefore, potential differences in medication use or additional care among groups cannot be excluded and represent an important limitation when interpreting the pain findings. Regression to the mean is another potential explanation; however, the available data do not strongly support this interpretation. Baseline pain intensity was highly comparable among groups (MEPG: 4.20, DEPG: 4.10, CEPG: 4.10; F(2, 27) = 0.079, *p* = 0.924), indicating similar potential for regression across groups. Furthermore, the association between baseline pain intensity and pain reduction was comparable among groups (r = −0.62 to −0.83). Therefore, regression to the mean alone is unlikely to explain the additional pain reduction observed in the CEPG. Nevertheless, these findings should be interpreted cautiously. The post-intervention pain score in the CEPG was 0.90, with three participants reporting no pain and eight reporting pain scores ≤ 1, resulting in clustering near the lower boundary of the scale. Thus, floor effects may have influenced the magnitude of observed differences. Furthermore, because participants with pain intensity ≥5 were excluded, the sample represented individuals with relatively mild chronic low back pain, and whether similar patterns would occur in patients with moderate or severe symptoms remains unknown.

In summary, the pain-related findings do not support the superiority of MedX lumbar extension or deadlift exercises over core stabilization exercises alone. Rather, they suggest that substantial pain improvement may be achievable through core stabilization exercise alone within an 8-week supervised program. Importantly, because this study used an add-on design in which all groups received the same core stabilization intervention, the primary question was not whether each exercise was effective in isolation but whether adding specific resistance-based exercises provided additional benefits beyond an established exercise program. Detecting such incremental effects requires larger sample sizes than those typically used in pilot studies. For example, previous research using a similar additive design identified between-group differences with approximately 30 participants per group [21], whereas the present study included only 10 participants per group. Therefore, the absence of clear between-group differences should not be interpreted as evidence of no additive effect but rather as preliminary findings requiring confirmation in adequately powered randomized controlled trials.

### 4.1. Implications for the Design of Future Definitive Trials

One of the primary contributions of a pilot study is the estimation of outcome variability to inform sample size calculations for a future definitive trial. In this study, the pooled standard deviations of change scores ranged from 36.86 to 41.54 ft-lbs across different lumbar extensor strength angles, 4.85 points for ADLs, and 1.02 points for pain intensity. Using these estimates, preliminary sample size calculations were performed for the future definitive trial. When the between-group effect sizes observed in the present pilot study were directly applied, the estimated sample sizes varied substantially across outcomes, ranging from 24 to 279 participants in total. This large variation is expected because effect size estimates derived from small pilot samples are highly susceptible to sampling variability and may not provide stable estimates of the true intervention effect. Therefore, assuming a conservative moderate effect size (Cohen’s f = 0.25), a three-group comparison with an alpha level of 0.05 and 80% statistical power would require approximately 53 participants per group (159 participants in total). Considering an anticipated 20% dropout rate, approximately 201 participants would need to be recruited. These estimates indicate that the present pilot study (total *n* = 30) was not adequately powered to detect small-to-moderate between-group differences. Accordingly, the limited number of significant between-group findings observed in this study should be interpreted cautiously.

Based on methodological issues identified during the conduct of this pilot study, several recommendations should be considered when designing future definitive trials. First, regarding participant selection and allocation, eligibility criteria should clearly define the target population through systematic screening for specific spinal pathologies using appropriate clinical assessments and imaging procedures. Future trials should also consider including participants of different sexes and age groups to evaluate potential effect modification by demographic characteristics. Furthermore, stratified randomization based on baseline functional impairment or symptom severity may help minimize imbalance between groups.

Second, regarding trial registration and allocation procedures, primary outcomes should be prespecified, and the study protocol and statistical analysis plan should be registered before participant recruitment. Random sequence generation, participant enrollment, and group allocation should be performed by independent personnel. Allocation concealment should be ensured through appropriate methods, such as sequentially numbered opaque sealed envelopes or centralized allocation systems, and all procedures should be prospectively documented.

Third, regarding intervention standardization, future studies should carefully control exercise volume across groups to isolate the specific additive effects of the intervention being investigated. Predefined criteria for progression, reassessment schedules for exercise intensity, and standardized reporting of exercise prescription variables, including rest intervals and movement velocity, should be incorporated into the protocol to improve intervention reproducibility.

Fourth, regarding measurement procedures, multiple maximal contractions at each testing angle should be considered to improve measurement reliability by allowing calculation of mean values. Alternatively, a separate familiarization session should be included before baseline testing. In addition, training and assessment equipment should be separated whenever possible to minimize potential learning effects.

Fifth, regarding outcome assessment, future studies should incorporate biopsychosocial measures, including fear of movement, depressive symptoms, and balance ability, alongside muscle strength and pain outcomes to better understand mechanisms underlying functional improvement. Intervention acceptability should also be assessed using validated structured questionnaires, and prospective adverse event monitoring procedures should be incorporated to improve safety evaluation.

Sixth, regarding potential confounding factors, particularly when pain intensity is selected as a primary outcome, analgesic use and concomitant treatments should be systematically monitored throughout the intervention period. These variables should be considered as potential covariates or evaluated as secondary outcomes reflecting changes in treatment requirements.

### 4.2. Limitations

This study has some limitations that should be considered when interpreting its findings. First, regarding study design and prior planning, the protocol and statistical analysis plan were not prospectively registered before participant recruitment. Clinical trial registration was conducted retrospectively on 7 June 2026, approximately 20 months after completion of data collection. Prospective registration helps reduce the potential for selective reporting by requiring pre-specification of outcomes and analytical approaches before results become available. In this study, the primary outcome was not designated a priori, post hoc pairwise comparisons were not pre-specified, and feasibility progression criteria were established retrospectively. Furthermore, the procedure for generating the randomization sequence, the personnel responsible for participant allocation, and whether allocation concealment was implemented were not documented at the time of the study and cannot be verified retrospectively. Therefore, the possibility of selection bias due to inadequate or undocumented allocation procedures cannot be excluded.

Second, regarding sampling and generalizability, this pilot study included a small sample of 30 participants (10 per group). Based on variability estimates obtained from the present data, a substantially larger sample size (approximately 53 participants per group) would likely be required to detect moderate between-group differences in a future definitive trial. In addition, participants were restricted to men aged 30–39 years with relatively mild low back pain (pain intensity < 5) recruited from a single exercise facility in one geographic region. Therefore, the findings may not be generalizable to women, other age groups, individuals with greater symptom severity, or clinical populations with different characteristics. Moreover, because specific spinal pathologies were not systematically excluded through imaging examinations or medical diagnoses, the participants should not be interpreted as representing a strictly defined population of non-specific chronic low back pain.

Third, regarding intervention and measurement procedures, although all groups performed the same core stabilization program, total exercise duration and volume differed between groups because MEPG and DEPG additionally performed the main exercises (approximately 45 min per session) whereas CEPG performed only the common program (approximately 35 min per session). Therefore, the independent effects of the additional exercises cannot be completely separated from the influence of increased exercise exposure. Furthermore, because the CEPG served as an active comparison group rather than a non-exercise control group, improvements observed across all groups may have been partly attributable to the common core stabilization program, limiting the ability to determine the unique effects of each additional intervention. Exercise intensity was adjusted according to the supervisor’s clinical judgment without predefined progression criteria, and systematic reassessment of training loads was not conducted during the 8-week intervention period. In addition, rest intervals between sets and movement velocities were not specified in advance or recorded, limiting intervention reproducibility. Regarding outcome assessment, maximal isometric contractions were performed only once at each angle, preventing estimation of within-participant measurement variability. A separate familiarization session was also not conducted before baseline assessment. Furthermore, because the MedX equipment was used for both training and outcome assessment, potential learning effects cannot be excluded. Intervention acceptability was not evaluated using a validated structured questionnaire, and prospective procedures for adverse event monitoring and documentation were not established. In addition, analgesic use and concomitant treatments during the intervention period were not systematically recorded or controlled, which represents an important potential confounder given that pain intensity was a self-reported outcome. The possibility of floor effects should also be considered because post-intervention pain scores in the CEPG were clustered near the lower end of the scale. Finally, mechanisms underlying changes in functional impairment could not be fully explored because relevant biopsychosocial factors, including kinesiophobia, depressive symptoms, and balance ability, were not assessed.

Fourth, regarding statistical considerations and interpretation, the initial sample size calculation assumed a large effect size (f = 0.50) without prior empirical justification, which may have resulted in an underestimation of the required sample size. Furthermore, repeated-measures ANOVA was conducted separately for nine exploratory outcomes, increasing the potential risk of Type I error inflation. Although multiple comparison adjustments were not applied in the primary analyses due to the exploratory nature of the pilot study, the findings should be interpreted primarily through effect estimates and confidence intervals rather than statistical significance alone. For ADLs, baseline differences were observed between groups, and the possibility of chance imbalance or selection bias related to undocumented allocation procedures cannot be excluded. Because the group with greater baseline impairment demonstrated larger reductions, regression to the mean may also have contributed to the observed pattern. In addition, the assumption of homogeneity of variance was violated for some outcome variables, which may have influenced the robustness of certain statistical comparisons.

Fifth, regarding uncontrolled factors, participants’ habitual exercise participation and previous physical activity levels were not assessed or controlled before the intervention. Differences in prior exercise exposure may have influenced adaptation to the exercise programs and should be considered in future studies.

## 5. Conclusions

In this pilot study, all three 8-week exercise programs for men in their 30s with chronic low back pain demonstrated high feasibility, with complete retention and attendance compliance and no reported intervention-related adverse events. These findings support the feasibility of proceeding to a future fully powered randomized controlled trial. Regarding the secondary exploratory outcomes, lumbar extensor strength, activities of daily living, and pain intensity improved significantly over time in all three groups. However, this study did not provide evidence that adding MedX lumbar extension exercises or deadlift exercises to a common core stabilization program resulted in additional benefits. No significant between-group differences were observed in lumbar extensor strength across any tested angles, and the apparent between-group differences in activities of daily living were not maintained after adjustment for baseline imbalance. In contrast, the greatest reduction in pain intensity was observed in the group performing core stabilization exercises alone. Therefore, these findings should not be interpreted as providing clinical recommendations regarding the superiority of any specific exercise approach. Rather, the preliminary effect estimates and variability parameters obtained from this pilot study should be used to inform the design of future adequately powered randomized controlled trials incorporating prospective registration, appropriate allocation procedures, and stratified randomization.

## Figures and Tables

**Table 1:** Baseline characteristics of the participants (*n* = 30).

Variable	MEPG (*n* = 10)	DEPG (*n* = 10)	CEPG (*n* = 10)	F (2, 27)	*p*	ηp^2^
Demographic and physical characteristics						
Age (years)	35.50 ± 2.80	33.90 ± 2.56	35.00 ± 3.71	0.714	0.499	0.050
Height (cm)	176.40 ± 3.63	170.30 ± 6.65	175.80 ± 3.52	4.751	0.016	0.260
Weight (kg)	71.02 ± 5.26	70.68 ± 7.69	70.68 ± 10.40	0.006	0.994	0.000
Body mass index (kg/m^2^)	22.81 ± 1.38	24.42 ± 2.39	22.95 ± 3.93	1.028	0.370	0.071
Outcome variables at baseline						
Extensor strength, 0° (ft-lbs)	102.50 ± 50.45	101.90 ± 50.90	83.10 ± 26.72	0.631	0.540	0.045
Extensor strength, 12° (N)	136.80 ± 44.12	113.40 ± 45.02	114.20 ± 46.22	0.867	0.432	0.060
Extensor strength, 24° (N)	157.10 ± 44.54	130.70 ± 57.51	127.40 ± 51.15	1.005	0.379	0.069
Extensor strength, 36° (N)	168.50 ± 41.51	148.40 ± 59.94	137.60 ± 43.95	1.018	0.375	0.070
Extensor strength, 48° (N)	175.70 ± 44.14	163.70 ± 65.14	151.00 ± 39.08	0.593	0.560	0.042
Extensor strength, 60° (N)	185.90 ± 47.49	166.60 ± 61.31	159.30 ± 41.78	0.730	0.491	0.051
Extensor strength, 72° (N)	185.40 ± 45.34	182.30 ± 62.55	167.70 ± 39.68	0.355	0.704	0.026
Oswestry Disability Index (points)	26.60 ± 6.40 (53.20 ± 12.80%)	18.40 ± 4.88 (36.80 ± 9.76%)	25.40 ± 5.66 (50.80 ± 11.32%)	6.079	0.007	0.310
Pain intensity, NRS (0–10)	4.20 ± 0.63	4.10 ± 0.74	4.10 ± 0.57	0.079	0.924	0.006

MEPG: MedX Exercise Program Group; DEPG: Deadlift Exercise Program Group; CEPG: Core Exercise Program Group; NRS: numeric rating scale; ηp^2^: partial eta squared. Values are mean ± standard deviation. Group homogeneity was examined using one-way analysis of variance. Higher Oswestry Disability Index scores indicate greater disability. Baseline Oswestry Disability Index scores differed significantly among groups; accordingly, analysis of covariance with the baseline value as covariate was additionally performed for this outcome.

**Table 2:** Structure of the MedX exercise program.

Phase	Classification	Exercise	Repetition or Time
MedX (1–8 weeks)	Warm-up (stretching)	Quadriceps	10 s 2 sets/15 min
		Hamstring	
		Iliopsoas	
		Piriformis	
		Glutes	
	Strengthening	MedX lumbar extension	15 reps 3 sets/10 min
	Additional exercise	Supine abdominal bracing	10 s 20 reps
		Standing abdominal bracing	
		Dead bug	15 reps 2 sets/15 min
		Birddog	
		Crunch	
		Leg raises	
	Cool-down	Cycling	5 min

The prescribed intensity was maintained throughout the 8-week period, with individual adjustments made by the supervising instructor. No predefined progression criteria were applied.

**Table 3:** Structure of the deadlift exercise program.

Phase	Classification	Exercise	Repetition or Time
Deadlift (1–8 weeks)	Warm-up (stretching)	Quadriceps	10 s 2 sets/15 min
		Hamstring	
		Iliopsoas	
		Piriformis	
		Glutes	
	Strengthening	Romanian deadlift	15 reps 3 sets/10 min
	Additional exercise	Supine abdominal bracing	10 s 20 reps
		Standing abdominal bracing	
		Dead bug	15 reps 2 sets/15 min
		Birddog	
		Crunch	
		Leg raises	
	Cool-down	Cycling	5 min

The prescribed intensity was maintained throughout the 8-week period, with individual adjustments made by the supervising instructor. No predefined progression criteria were applied.

**Table 4:** Structure of the core exercise program.

Phase	Classification	Exercise	Repetition or Time
Core (1–8 weeks)	Warm-up (stretching)	Quadriceps	10 s 2 sets/15 min
		Hamstring	
		Iliopsoas	
		Piriformis	
		Glutes	
	Additional exercise	Supine abdominal bracing	10 s 20 reps
		Standing abdominal bracing	
		Dead bug	15 reps 2 sets/15 min
		Birddog	
		Crunch	
		Leg raises	
	Cool-down	Cycling	5 min

The prescribed intensity was maintained throughout the 8-week period, with individual adjustments made by the supervising instructor. No predefined progression criteria were applied.

**Table 5:** Changes in lumbar extensor strength at 0°.

Variable	MEPG (*n* = 10)	DEPG (*n* = 10)	CEPG (*n* = 10)	Source	F	df	*p*	ηp^2^ (95% CI)
Pre- intervention	102.50 ± 37.03	101.90 ± 50.90	83.10 ± 42.67	Time	68.894	1, 27	<0.001	0.718 (0.494–0.813)
Post- intervention	187.10 ± 60.07	141.20 ± 40.77	138.40 ± 35.43	Group	1.821	2, 27	0.181	0.119 (0.000–0.317)
Mean change (95% CI)	84.60 (48.40 to 120.80)	39.30 (18.70 to 59.90)	55.30 (29.80 to 80.80)	Time × group ^b^	3.397	2, 27	0.048	0.201 (0.000–0.405)
Change (%)	+82.54	+38.57	+66.55	Baseline homogeneity ^c^	0.631	2, 27	0.540	0.045
Within-group *p* ^a^	0.002	0.006	0.003					
Cohen’s dz	1.67	1.36	1.55					

MEPG: MedX Exercise Program Group; DEPG: Deadlift Exercise Program Group; CEPG: Core Exercise Program Group; CI: confidence interval; ηp^2^: partial eta squared; dz: Cohen’s d for paired samples. Lumbar extensor strength was measured in ft-lbs. Values are mean ± standard deviation unless otherwise indicated. ^a^ Paired *t*-test, Bonferroni-adjusted for three groups. ^b^ Pairwise comparisons of change scores (Bonferroni): MEPG vs. DEPG mean difference 45.30, *p* = 0.073; MEPG vs. CEPG 29.30, *p* = 0.455; DEPG vs. CEPG −16.00, *p* = 0.852. No pairwise comparison reached significance. ^c^ One-way analysis of variance on pre-intervention values. This study was exploratory; *p* values are descriptive and were not adjusted for multiple outcomes.

**Table 6:** Changes in lumbar extensor strength at 12°.

Variable	MEPG (*n* = 10)	DEPG (*n* = 10)	CEPG (*n* = 10)	Source	F	df	*p*	ηp^2^ (95% CI)
Pre- intervention	136.80 ± 44.12	113.40 ± 45.02	114.20 ± 46.22	Time	75.297	1, 27	<0.001	0.736 (0.521–0.824)
Post- intervention	212.50 ± 62.57	175.40 ± 52.81	162.00 ± 34.75	Group	1.952	2, 27	0.162	0.126 (0.000–0.326)
Mean change (95% CI)	75.70 (34.46 to 116.94)	62.00 (48.12 to 75.88)	47.80 (26.70 to 68.90)	Time × group	1.278	2, 27	0.295	0.086 (0.000–0.275)
Change (%)	+55.34	+54.67	+41.86	Baseline homogeneity ^b^	0.867	2, 27	0.432	0.060
Within-group *p* ^a^	0.007	<0.001	0.002					
Cohen’s dz	1.31	3.19	1.62					

MEPG: MedX Exercise Program Group; DEPG: Deadlift Exercise Program Group; CEPG: Core Exercise Program Group; CI: confidence interval; ηp^2^: partial eta squared; dz: Cohen’s d for paired samples. Lumbar extensor strength was measured in ft-lbs. Values are mean ± standard deviation unless otherwise indicated. ^a^ Paired *t*-test, Bonferroni-adjusted for three groups. ^b^ One-way analysis of variance on pre-intervention values. This study was exploratory; *p* values are descriptive and were not adjusted for multiple outcomes.

**Table 7:** Changes in lumbar extensor strength at 24°.

Variable	MEPG (*n* = 10)	DEPG (*n* = 10)	CEPG (*n* = 10)	Source	F	df	*p*	ηp^2^ (95% CI)
Pre- intervention	157.10 ± 44.54	130.70 ± 57.51	127.40 ± 51.15	Time	85.599	1, 27	<0.001	0.760 (0.560–0.840)
Post- intervention	233.90 ± 64.20	194.80 ± 51.64	186.70 ± 38.46	Group	1.868	2, 27	0.174	0.122 (0.000–0.320)
Mean change (95% CI)	76.80 (36.52 to 117.08)	64.10 (47.61 to 80.59)	59.30 (36.91 to 81.69)	Time × group	0.524	2, 27	0.598	0.037 (0.000–0.193)
Change (%)	+48.89	+49.04	+46.55	Baseline homogeneity ^b^	1.005	2, 27	0.379	0.069
Within-group *p* ^a^	0.006	<0.001	<0.001					
Cohen’s dz	1.36	2.78	1.89					

MEPG: MedX Exercise Program Group; DEPG: Deadlift Exercise Program Group; CEPG: Core Exercise Program Group; CI: confidence interval; ηp^2^: partial eta squared; dz: Cohen’s d for paired samples. Lumbar extensor strength was measured in ft-lbs. Values are mean ± standard deviation unless otherwise indicated. ^a^ Paired *t*-test, Bonferroni-adjusted for three groups. ^b^ One-way analysis of variance on pre-intervention values. This study was exploratory; *p* values are descriptive and were not adjusted for multiple outcomes.

**Table 8:** Changes in lumbar extensor strength at 36°.

Variable	MEPG (*n* = 10)	DEPG (*n* = 10)	CEPG (*n* = 10)	Source	F	df	*p*	ηp^2^ (95% CI)
Pre- intervention	168.50 ± 41.51	148.40 ± 59.94	137.60 ± 43.95	Time	94.418	1, 27	<0.001	0.778 (0.589–0.852)
Post- intervention	248.30 ± 66.28	208.70 ± 47.71	198.50 ± 41.76	Group	1.945	2, 27	0.163	0.126 (0.000–0.325)
Mean change (95% CI)	79.80 (44.61 to 114.99)	60.30 (37.60 to 83.00)	60.90 (40.01 to 81.79)	Time × group	0.862	2, 27	0.434	0.060 (0.000–0.235)
Change (%)	+47.36	+40.63	+44.26	Baseline homogeneity ^b^	1.018	2, 27	0.375	0.070
Within-group *p* ^a^	0.002	<0.001	<0.001					
Cohen’s dz	1.62	1.90	2.09					

MEPG: MedX Exercise Program Group; DEPG: Deadlift Exercise Program Group; CEPG: Core Exercise Program Group; CI: confidence interval; ηp^2^: partial eta squared; dz: Cohen’s d for paired samples. Lumbar extensor strength was measured in ft-lbs. Values are mean ± standard deviation unless otherwise indicated. ^a^ Paired *t*-test, Bonferroni-adjusted for three groups. ^b^ One-way analysis of variance on pre-intervention values. This study was exploratory; *p* values are descriptive and were not adjusted for multiple outcomes.

**Table 9:** Changes in lumbar extensor strength at 48°.

Variable	MEPG (*n* = 10)	DEPG (*n* = 10)	CEPG (*n* = 10)	Source	F	df	*p*	ηp^2^ (95% CI)
Pre- intervention	175.70 ± 44.14	163.70 ± 65.14	151.00 ± 39.08	Time	82.125	1, 27	<0.001	0.753 (0.548–0.835)
Post- intervention	259.50 ± 66.82	221.40 ± 47.41	207.40 ± 39.40	Group	1.677	2, 27	0.206	0.110 (0.000–0.307)
Mean change (95% CI)	83.80 (49.70 to 117.90)	57.70 (28.50 to 86.90)	56.40 (35.79 to 77.01)	Time × group	1.503	2, 27	0.240	0.100 (0.000–0.294)
Change (%)	+47.69	+35.25	+37.35	Baseline homogeneity ^b^	0.593	2, 27	0.560	0.042
Within-group *p* ^a^	0.001	0.005	<0.001					
Cohen’s dz	1.76	1.41	1.96					

MEPG: MedX Exercise Program Group; DEPG: Deadlift Exercise Program Group; CEPG: Core Exercise Program Group; CI: confidence interval; ηp^2^: partial eta squared; dz: Cohen’s d for paired samples. Lumbar extensor strength was measured in ft-lbs. Values are mean ± standard deviation unless otherwise indicated. ^a^ Paired *t*-test, Bonferroni-adjusted for three groups. ^b^ One-way analysis of variance on pre-intervention values. This study was exploratory; *p* values are descriptive and were not adjusted for multiple outcomes.

**Table 10:** Changes in lumbar extensor strength at 60°.

Variable	MEPG (*n* = 10)	DEPG (*n* = 10)	CEPG (*n* = 10)	Source	F	df	*p*	ηp^2^ (95% CI)
Pre- intervention	185.90 ± 47.49	166.60 ± 61.31	159.30 ± 41.78	Time	118.201	1, 27	<0.001	0.814 (0.651–0.876)
Post- intervention	276.50 ± 72.60	236.60 ± 53.72	218.20 ± 38.08	Group	1.855	2, 27	0.176	0.121 (0.000–0.319)
Mean change (95% CI)	90.60 (55.79 to 125.41)	70.00 (44.38 to 95.62)	58.90 (44.15 to 73.65)	Time × group	1.904	2, 27	0.168	0.124 (0.000–0.323)
Change (%)	+48.74	+42.02	+36.97	Baseline homogeneity ^b^	0.730	2, 27	0.491	0.051
Within-group *p* ^a^	<0.001	<0.001	<0.001					
Cohen’s dz	1.86	1.95	2.86					

MEPG: MedX Exercise Program Group; DEPG: Deadlift Exercise Program Group; CEPG: Core Exercise Program Group; CI: confidence interval; ηp^2^: partial eta squared; dz: Cohen’s d for paired samples. Lumbar extensor strength was measured in ft-lbs. Values are mean ± standard deviation unless otherwise indicated. ^a^ Paired *t*-test, Bonferroni-adjusted for three groups. ^b^ One-way analysis of variance on pre-intervention values. This study was exploratory; *p* values are descriptive and were not adjusted for multiple outcomes.

**Table 11:** Changes in lumbar extensor strength at 72°.

Variable	MEPG (*n* = 10)	DEPG (*n* = 10)	CEPG (*n* = 10)	Source	F	df	*p*	ηp^2^ (95% CI)
Pre- intervention	185.40 ± 45.34	182.30 ± 62.55	167.70 ± 39.68	Time	93.257	1, 27	<0.001	0.775 (0.585–0.851)
Post- intervention	284.40 ± 79.72	248.10 ± 58.49	222.60 ± 34.58	Group	1.488	2, 27	0.244	0.099 (0.000–0.293)
Mean change (95% CI)	99.00 (58.09 to 139.91)	65.80 (37.64 to 93.96)	54.90 (41.41 to 68.39)	Time × group	3.058	2, 27	0.064	0.185 (0.000–0.389)
Change (%)	+53.40	+36.09	+32.74	Baseline homogeneity ^b^	0.355	2, 27	0.704	0.026
Within-group *p* ^a^	0.001	0.002	<0.001					
Cohen’s dz	1.73	1.67	2.91					

MEPG: MedX Exercise Program Group; DEPG: Deadlift Exercise Program Group; CEPG: Core Exercise Program Group; CI: confidence interval; ηp^2^: partial eta squared; dz: Cohen’s d for paired samples. Lumbar extensor strength was measured in ft-lbs. Values are mean ± standard deviation unless otherwise indicated. ^a^ Paired *t*-test, Bonferroni-adjusted for three groups. ^b^ One-way analysis of variance on pre-intervention values. This study was exploratory; *p* values are descriptive and were not adjusted for multiple outcomes.

**Table 12:** Changes in the ability to perform ADLs.

Variable	MEPG (*n* = 10)	DEPG (*n* = 10)	CEPG (*n* = 10)	Source	F	df	*p*	ηp^2^ (95% CI)
Pre- intervention	26.60 ± 6.40 (53.20 ± 12.80%)	18.40 ± 4.88 (36.80 ± 9.76%)	25.40 ± 5.66 (50.80 ± 11.32%)	Time	261.816	1, 27	<0.001	0.907 (0.818–0.938)
Post- intervention	9.80 ± 1.75 (19.60 ± 3.50%)	9.00 ± 4.24 (18.00 ± 8.49%)	8.60 ± 1.90 (17.20 ± 3.79%)	Group	3.782	2, 27	0.036	0.219 (0.000–0.422)
Mean change (95% CI)	−16.80 (−20.51 to −13.09)	−9.40 (−11.43 to −7.37)	−16.80 (−21.08 to −12.52)	Time × group	7.754	2, 27	0.002	0.365 (0.067–0.548)
Change (%)	−63.16	−51.09	−66.14	Group (ANCOVA) ^c^	1.574	2, 26	0.226	0.108
Within-group *p* ^a^	<0.001	<0.001	<0.001	Baseline homogeneity ^d^	6.079	2, 27	0.007	0.310
Cohen’s dz	−3.24	−3.31	−2.81					
Adjusted mean ^c,b^	8.98	10.33	8.09					

MEPG: MedX Exercise Program Group; DEPG: Deadlift Exercise Program Group; CEPG: Core Exercise Program Group; CI: confidence interval; ηp^2^: partial eta squared; dz: Cohen’s d for paired samples; ANCOVA: analysis of covariance. The ability to perform activities of daily living was assessed using the Korean version of the Oswestry Disability Index. Values are mean ± standard deviation unless otherwise indicated; higher scores indicate greater disability. Values in parentheses are ODI percentage scores (raw score ÷ 50 × 100). All analyses were performed on raw scores. ^a^ Paired *t*-test, Bonferroni-adjusted for three groups. ^b^ Pairwise comparisons of ANCOVA-adjusted means (Bonferroni): MEPG vs. DEPG *p* = 0.276; MEPG vs. CEPG *p* = 0.575; DEPG vs. CEPG *p* = 0.414. ^c^ ANCOVA with the baseline value as covariate, performed because baseline scores differed significantly among groups. Covariate mean = 23.47. ^d^ One-way analysis of variance on pre-intervention values. This study was exploratory; *p* values are descriptive and were not adjusted for multiple outcomes.

**Table 13:** Changes in pain intensity (NRS).

Variable	MEPG (*n* = 10)	DEPG (*n* = 10)	CEPG (*n* = 10)	Source	F	df	*p*	ηp^2^ (95% CI)
Pre- intervention	4.20 ± 0.63	4.10 ± 0.74	4.10 ± 0.57	Time	152.488	1, 27	<0.001	0.850 (0.714–0.900)
Post- intervention	2.40 ± 0.70	2.20 ± 0.79	0.90 ± 0.74	Group	7.971	2, 27	0.002	0.371 (0.071–0.553)
Mean change (95% CI)	−1.80 (−2.46 to −1.14)	−1.90 (−2.61 to −1.19)	−3.20 (−4.01 to −2.39)	Time × group ^b^	5.861	2, 27	0.008	0.303 (0.028–0.497)
Change (%)	−42.86	−46.34	−78.05	Baseline homogeneity ^c^	0.079	2, 27	0.924	0.006
Within-group *p* ^a^	<0.001	<0.001	<0.001					
Cohen’s dz	−1.96	−1.91	−2.82					

MEPG: MedX Exercise Program Group; DEPG: Deadlift Exercise Program Group; CEPG: Core Exercise Program Group; NRS: numeric rating scale; CI: confidence interval; ηp^2^: partial eta squared; dz: Cohen’s d for paired samples. Pain intensity was assessed using an 11-point numeric rating scale (0 = no pain, 10 = worst imaginable pain). Values are mean ± standard deviation unless otherwise indicated. ^a^ Paired *t*-test, Bonferroni-adjusted for three groups. ^b^ Pairwise comparisons of change scores (Bonferroni): CEPG vs. MEPG mean difference −1.40, *p* = 0.022, Hedges’ *g* = −1.30; CEPG vs. DEPG −1.30, *p* = 0.042, Hedges’ *g* = −1.17; DEPG vs. MEPG −0.10, *p* = 1.000, Hedges’ *g* = −0.10. ^c^ One-way analysis of variance on pre-intervention values. This study was exploratory; *p* values are descriptive and were not adjusted for multiple outcomes.

## Data Availability

The anonymized raw data containing all outcome variables measured in this study are available from the corresponding author upon reasonable request. The data were not deposited in a public repository due to the scope of participant consent and ethical considerations related to data sharing. No measured outcome variables have been omitted from the reported analyses.

## Appendix A

**Table A1:** Sensitivity analysis for multiple testing across the nine outcome variables.

**(a) Group Effect**				
**Outcome**	**Unadjusted**	**Bonferroni**	**Holm**	**Benjamini–Hochberg**
0°	0.181	1.000	1.000	0.232
12°	0.162	1.000	1.000	0.232
24°	0.174	1.000	1.000	0.232
36°	0.163	1.000	1.000	0.232
48°	0.206	1.000	1.000	0.232
60°	0.176	1.000	1.000	0.232
72°	0.244	1.000	1.000	0.244
ODI	0.036	0.321	0.285	0.160
Pain intensity	**0.002**	**0.017**	**0.017**	**0.017**
**(** **b** **) Time × Group Interaction**				
**Outcome**	**Unadjusted**	**Bonferroni**	**Holm**	**Benjamini–Hochberg**
0°	0.048	0.435	0.338	0.143
12°	0.295	1.000	0.962	0.379
24°	0.598	1.000	0.962	0.598
36°	0.434	1.000	0.962	0.488
48°	0.240	1.000	0.962	0.361
60°	0.168	1.000	0.842	0.303
72°	0.064	0.572	0.381	0.143
Oswestry Disability Index	**0.002**	**0.020**	**0.020**	**0.020**
Pain intensity	0.008	0.069	0.062	0.035

Values are *p* values. Corrections were applied within each family of nine outcomes. The main effect of time is not shown because it remained significant at *p* < 0.001 for all nine outcomes under every correction method. Bolded values indicate effects that remained significant after Bonferroni and Holm correction.

**Table A2:** Post hoc comparisons for outcomes with a significant time × group interaction.

**(a) Within-Group Change (Pre- vs. Post-Intervention)**				
**Outcome**	**Group**	**Mean Change (95% CI)**	***p*** **^a^**	**Cohen’s dz**
0°	MEPG	84.60 (48.40 to 120.80)	0.002	1.67
	DEPG	39.30 (18.70 to 59.90)	0.006	1.36
	CEPG	55.30 (29.80 to 80.80)	0.003	1.55
Oswestry Disability Index	MEPG	−16.80 (−20.51 to −13.09)	<0.001	−3.24
	DEPG	−9.40 (−11.43 to −7.37)	<0.001	−3.31
	CEPG	−16.80 (−21.08 to −12.52)	<0.001	−2.81
Pain	MEPG	−1.80 (−2.44 to −1.16)	<0.001	−2.01
	DEPG	−1.90 (−2.60 to −1.20)	<0.001	−1.93
	CEPG	−3.20 (−3.98 to −2.42)	<0.001	−2.93
**(b) Between-Group Comparisons**				
**Outcome**	**Comparison**	**Change Score: Difference (*p*) ^b^**	**Post-Intervention: Difference (*p*) ^b^**	
0°	MEPG vs. DEPG	45.30 (0.073)	45.90 (0.183)	
	MEPG vs. CEPG	29.30 (0.455)	48.70 (0.121)	
	DEPG vs. CEPG	−16.00 (0.852)	2.80 (1.000)	
Oswestry Disability Index	MEPG vs. DEPG	−7.40 (0.276) ^c^	0.80 (1.000)	
	MEPG vs. CEPG	0.00 (0.575) ^c^	1.20 (0.477)	
	DEPG vs. CEPG	7.40 (0.414) ^c^	0.40 (1.000)	
Pain	MEPG vs. DEPG	0.10 (1.000)	0.20 (1.000)	
	MEPG vs. CEPG	**1.40 (0.022)**	**1.50 (<0.001)**	
	DEPG vs. CEPG	**1.30 (0.042)**	**1.30 (0.004)**	

Only outcomes with a significant time × group interaction are shown (0° lumbar extensor strength, Oswestry Disability Index, and pain intensity). ^a^ Paired *t*-test, Bonferroni-adjusted for three groups. ^b^ Bonferroni-adjusted for three pairwise comparisons. ^c^ *p* values from pairwise comparisons of ANCOVA-adjusted means, because baseline ODI scores differed significantly among groups. Bolded values indicate statistically significant comparisons. Units: ft-lbs for extensor strength, points for ODI, 0–10 scale for pain intensity. MEPG: MedX Exercise Program Group; DEPG: Deadlift Exercise Program Group; CEPG: Core Exercise Program Group; CI: confidence interval; ODI: Oswestry Disability Index; NRS: numeric rating scale.
